# The value of systematic lymphadenectomy during debulking surgery in the treatment of ovarian cancer: a meta-analysis of randomized controlled trials

**DOI:** 10.1186/s13048-020-00653-4

**Published:** 2020-05-08

**Authors:** Qingqing Lin, Wenchao Liu, Song Xu, Juan Li, Jinyi Tong

**Affiliations:** 1 Department of Gynecology, Affiliated Hangzhou First People’s Hospital, Zhejiang University School of Medicine, 261 Huansha Rd, Hangzhou City, 310006 Zhejiang Province China; 2 Department of Neurosurgery, First Affiliated Hospital, School of Medicine, Zhejiang University, Hangzhou, 310003 Zhejiang China

**Keywords:** Systematic lymphadenectomy, Ovarian cancer, Overall survival, Progression-free survival, Complications, Meta-analysis

## Abstract

**Background:**

The therapeutic value of systematic lymphadenectomy during debulking surgery for ovarian cancer remains controversial. We conduct this meta-analysis to evaluate the significance of systematic lymphadenectomy in patients treated with optimal cytoreduction for ovarian cancer.

**Method:**

The PubMed, Medline, Embase, Cochrane Library and Web of Science databases were searched up to October 2019. Only English-language publications of randomized controlled trials (RCTs) that investigated the role of systematic lymphadenectomy in patients with ovarian cancer were selected for this analysis. For overall survival (OS) and progression-free survival (PFS), pooled hazard ratios (HR) with 95% confidence intervals (CIs) were calculated; for complications rate, we calculated pooled risk ratio (RR) with 95% confidence interval (CI). Statistical heterogeneity was assessed using both the I^2^ and chi-square tests. In cases of I^2^ being larger than 50%, a random-effect model was used, otherwise a fixed-effect model was used.

**Results:**

Four RCTs involving 1607 patients were included in the present analysis. There was no difference in OS between systematic lymphadenectomy and unsystematic lymphadenectomy (HR = 1.00; 95% CI = 0.94, 1.07; *p* = 0.90). Similarly, no significant difference was observed in PFS between these two groups (HR = 0.97; 95% CI = 0.87, 1.08; *p* = 0.62). And postoperative complications occurred more frequently in the systematic lymphadenectomy group (RR = 1.50; 95% CI = 1.34, 1.68; *p* < 0.00001).

**Conclusion:**

Systematic lymphadenectomy in patients with optimally cytoreduced ovarian cancer was not associated with longer overall or progression-free survival than unsystematic lymphadenectomy and was associated with a higher incidence of postoperative complications.

## Background

Ovarian cancer is the most lethal gynecologic malignancy and the eighth leading cause of cancer deaths among women worldwide [1]. Because of the absence of effective measures for early detection, it is often diagnosed at an advanced stage and the overall 5 year survival is only about 30% [2, 3]. The mainstay of treatment of ovarian cancer is primary surgery aimed at complete resection of all visible tumor followed by combination chemotherapy including platinum and paclitaxel [4]. Although most patients initially respond well to primary combined treatment, about 80% relapse within 5 years [5] and eventually die because of significant intraperitoneal or lymph node metastasis.

Lymphatic spread has been reported to be a common feature and an important prognostic factor in ovarian cancer. The rate of lymph node metastasis is 44 to 53% detected by systematic lymphadenectomy (SL) in patients with disease in all International Federation of Gynecology and Obstetrics (FIGO) stages [6, 7]. Although the association between pelvic or para-aortic lymph node metastasis and poor prognosis has been established [8], considerable debate has focused on whether these lymph nodes should be systematically removed during primary surgery.

The efficacy of SL on survival is controversial. Several retrospective studies have reported that SL is associated with improved survival [9–12]. However, other investigators have questioned the therapeutic efficacy of SL [13, 14]. In addition, the previous meta-analyses also have indicated that lymphadenectomy can provide a survival benefit in all-stage disease [15–17]. But their statistical methods didn’t employ hazards ratio (HR) and most of the studies included were retrospective whose data was not so reliable. Recently, the Lymphadenectomy in Ovarian Neoplasms (LION) trial, which was a famous and large RCT, showed that SL after maximal cytoreduction did not improve survival and may cause additional harm [18]. These results were entirely different from previous studies.

Therefore, we designed this meta-analysis including the new RCT to reevaluate the role of SL in ovarian cancer, and this is the first meta-analysis of all qualified relevant RCTs performed to date in which the postoperative complications are also analyzed.

## Methods

This systematic review and meta-analysis was conducted according to the guidelines of the Preferred Reporting Items for Systematic Reviews and Meta-Analyses (PRISMA) [19]. Because all analyses were performed using data from previously published studies, ethical approval and patient consent were unnecessary. Two reviewers independently performed the literature searches, data extraction, and quality assessment, and any disagreements were resolved by discussion.

### Literature search and selection criteria

The two reviewers systematically searched the PubMed, Medline, Embase, Cochrane Library, and Web of Science databases up to October 2019. The following terms were searched: Lymphadenectomy or lymph node dissection or lymph node or lymph node sampling AND ovarian cancer or ovarian tumor or ovarian neoplasm or ovarian carcinoma. Additionally, the reference lists of the identified articles and the “Related Articles” feature in PubMed were reviewed to maximize the probability of finding additional suitable papers. All English-language publications of RCTs that investigated the effects of SL in patients with ovarian cancer were included.

The exclusion criteria for the present study were as follows: (1) non-randomized clinical trials, (2) incomplete information for a quantitative analysis, (3) non-human models or non-English-language publications, or (4) no comparison between SL and unsystematic lymphadenectomy (USL). Two reviewers independently screened and excluded papers based on the abstracts using the inclusion and exclusion criteria; then, the full-text articles with potentially relevant abstracts were retrieved and independently assessed according to the inclusion and exclusion checklists. All disagreements were resolved by discussion until a consensus was reached; if this failed, a third reviewer (Jinyi Tong) was consulted.

### Data extraction

Two reviewers independently extracted the data from eligible primary studies and transferred them into a standard data extraction form. These data included the first author, year of publication, study design, number of participants in each group, participant age, clinical stage, definition of SL and USL, survival (OS and PFS) and complications. The primary outcome in the present meta-analysis was OS; the secondary outcome was PFS. For safety, we also examined complications rate after surgery.

### Quality assessment

The quality levels of the included studies were independently assessed by two reviewers, and any disagreement was resolved through discussion and consensus. Briefly, the Cochrane Collaboration tool [20] was used to assess the risk of bias with respect to the following: selection bias (random sequence generation and allocation concealment), attrition bias (incomplete outcome data), performance and detection bias (blinding of participants, personnel and outcome assessment), reporting bias (selective reporting), and other biases (other sources of bias).

### Statistical analysis

All data syntheses and analyses were performed using RevMan 5.3 software (Cochrane Collaboration; Oxford, UK). For OS and PFS, pooled hazard ratios (HR; SL vs. USL) with 95% confidence intervals (CIs) were calculated; for complications rate, we calculated pooled risk ratio (RR, SL vs. USL) with 95% CI. Statistical heterogeneity was assessed using both the I^2^ and chi-square tests. In cases of I^2^ being larger than 50%, a random-effect model was used, otherwise a fixed-effect model was used [21].

Sensitivity analyses were used to assess the stability of the results, and funnel plots were used to screen for potential publication biases.

## Results

### Overview

**Fig. 1:**
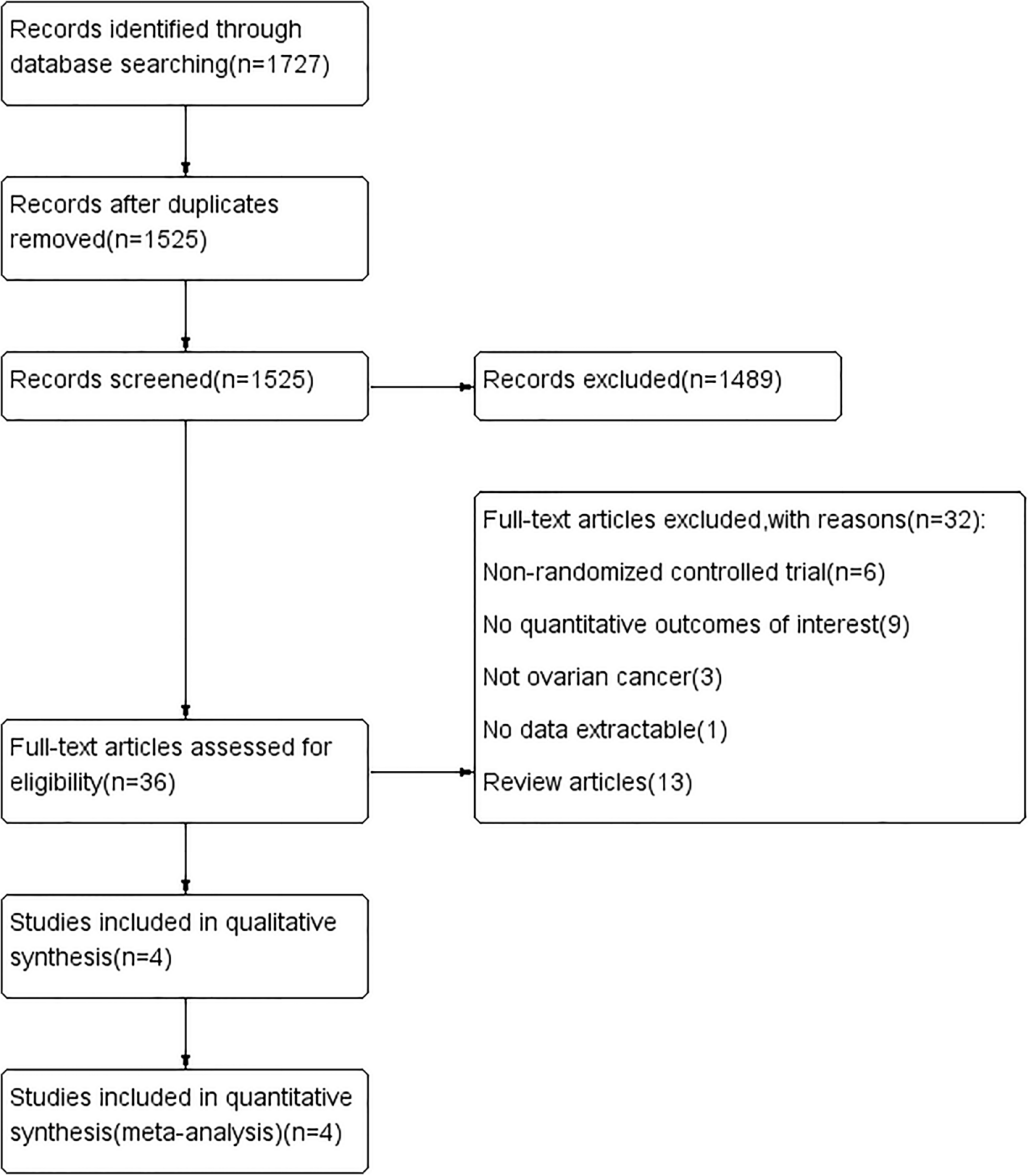
Flow diagram showing selection of the studies for this meta-analysis

Our search strategy identified 1727 articles, 1691 of which were excluded by the title and abstract screening processes. Of the remaining 36 articles, full texts were accessed and, ultimately, four RCTs met our inclusion criteria and were included in this review [18, 22–24]. Figure 1 presents a flow chart illustrating the above search process.

### Description of studies

The basic characteristics of the four studies are summarized in Table 1. These four articles are all RCTs [18, 22–24]. There are 808 patients in the SL group, and 799 patients in the USL group. The median age ranged from 50 to 60 years. Of the four trials, three studies [22–24] included both patients with macroscopically complete resection(R0) and those with residual tumors of up to 1 cm in diameter after surgery(R1), and the patients in one study [18] were macroscopically completely resected(R0). SL was defined as follows: (1) systematic pelvic and aortic lymphadenectomy [18]; (2) pelvic (≥20) and para-aortic (≥15) resected lymph nodes [23]; and (4) pelvic (≥25) and para-aortic (≥15) resected lymph nodes [22, 24]. And USL was defined as follows: (1) no lymphadenectomy [18]; (2) random removal of pelvic and para-aortic lymph nodes (sampling) [23]; and (3) removal of all macroscopic (≥1 cm) lymph nodes [22, 24].

**Table 1:** Characteristics of the studies included in the analysis

First author (publication year)	Study design	Histology type	FIGO stage	Debulking Surgery	Group	Number of patients	Median age (y)	Definition of SL and USL
Harter (2019) [18]	RCT	Epithelial ovarian cancer	IIB–IV	R0	SL	323	60	systematic pelvic and aortic lymphadenectomy
					USL	324	60	no lymphadenectomy
Maggioni (2006) [23]	RCT	Epithelial ovarian cancer	I–II	R1	SL	138	51	systematic pelvic (≥20) and aortic (≥15) lymphadenectomy
					USL	130	52	random removal of pelvic and para-aortic LNs (sampling)
Panici (2005) [22]	RCT	Epithelial ovarian cancer	IIIB–IV	R1	SL	189	53	systematic pelvic (≥25) and aortic (≥15) lymphadenectomy
					USL	195	56	bulky nodes only
Dell’ Anna (2012) [24]	RCT	Epithelial ovarian cancer	I–IV	R1	SL	158	50	systematic pelvic (≥25) and aortic (≥15) lymphadenectomy
					USL	150	52	bulky nodes only

*RCT* randomized controlled trial, *FIGO* International Federation of Gynecology and Obstetrics*, R0* no residual tumor, *R1* residual tumor < 1 cm, *SL* systematic lymphadenectomy, *USL* unsystematic lymphadenectomy

### Quality assessment

The quality assessments of the included RCTs are summarized in Table 2; the overall quality of the trials was determined to be good. Briefly, the randomization methods were described in all the four studies, and the allocation concealments was unclear in one study and high risk in one study, the blinding of outcome was unclear in two studies. In terms of incomplete outcome data and other bias, all four studies were considered to be low risk. Finally, the selective outcome reporting was unclear in one study.

**Table 2:** Quality assessments for the included randomized controlled studies using the Cochrane Collaboration’s tool

Study	Sequence generation	Allocation concealment	Blinding of outcome assessment	Incomplete outcome data	Selective reporting	Other bias
Harter (2019) [18]	Low risk	Low risk	Low risk	Low risk	Low risk	Low risk
Dell’ Anna (2012) [24]	Low risk	High risk	Unclear risk	Low risk	Low risk	Low risk
Maggioni (2006) [23]	Low risk	Unclear risk	Low risk	Low risk	Unclear risk	Low risk
Panici (2005) [22]	Low risk	Low risk	Unclear risk	Low risk	Low risk	Low risk

### Overall survival

**Fig. 2:**
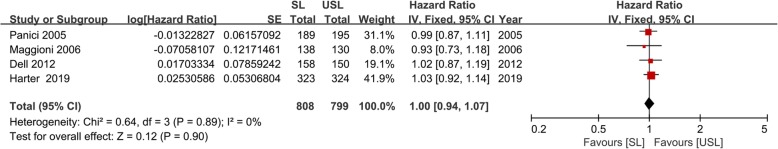
Forest plot for comparison of OS between SL and USL in all-stage disease. Results are shown by using a fixed-effect model with HR and 95% CIs

All four studies, with a total number of 1607 patients, were included in the meta-analysis for OS between the SL and USL groups in all-stage disease. A fixed-effect model of analysis was used, and the result indicated that there was no significant difference in OS between SL and USL groups (HR = 1.00; 95% CI = 0.94, 1.07; *p* = 0.90; Fig. 2). In addition, heterogeneity was not observed (I^2^ = 0%; *p* = 0.89) in this analysis.

### Progression-free survival

**Fig. 3:**
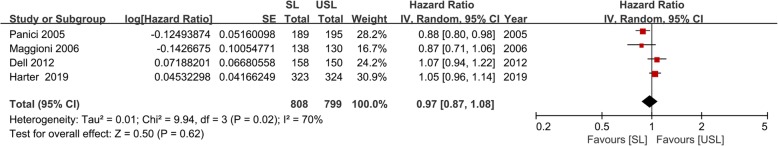
Forest plot for comparison of PFS between SL and USL in all-stage disease. Results are shown by using a random-effect model with HR and 95% CIs

Meta-analysis, including all four RCTs, assessing 1607 women, investigated PFS between the SL and USL groups in all-stage disease. A random-effect model of analysis was used, and it also showed no significant difference in PFS between the SL and USL groups (HR = 0.97; 95% CI = 0.87, 1.08; *p* = 0.62; Fig. 3), but heterogeneity was observed (I^2^ = 70%; *p* = 0.02).

### Postoperative complications

**Fig. 4:**
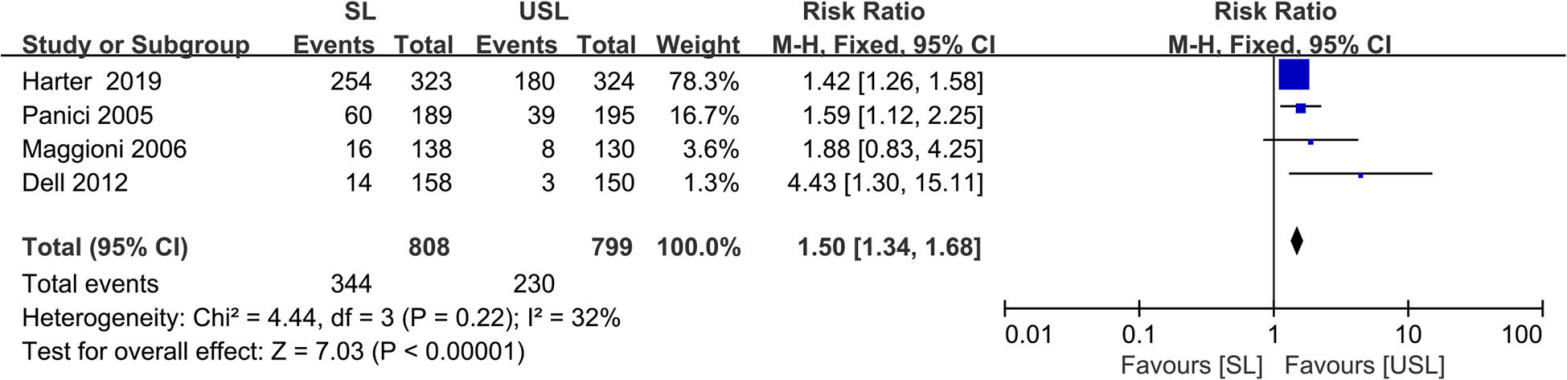
Forest plot of postoperative complications. Results are shown by using a fixed-effect model with RR and 95% CIs

Four RCTs all reported the number of postoperative complications in the SL and USL groups. Postoperative complications occurred in 42.6% of patients in the SL group and 28.8% of patients in the USL group. The pooled analysis revealed that postoperative complications occurred more frequently in the SL group (RR = 1.50; 95% CI = 1.34, 1.68; *p* < 0.00001; Fig. 4). There was little heterogeneity among the studies (I^2^ = 32%; *p* = 0.22).

### Sensitivity analysis and publication Bias

**Fig. 5:**
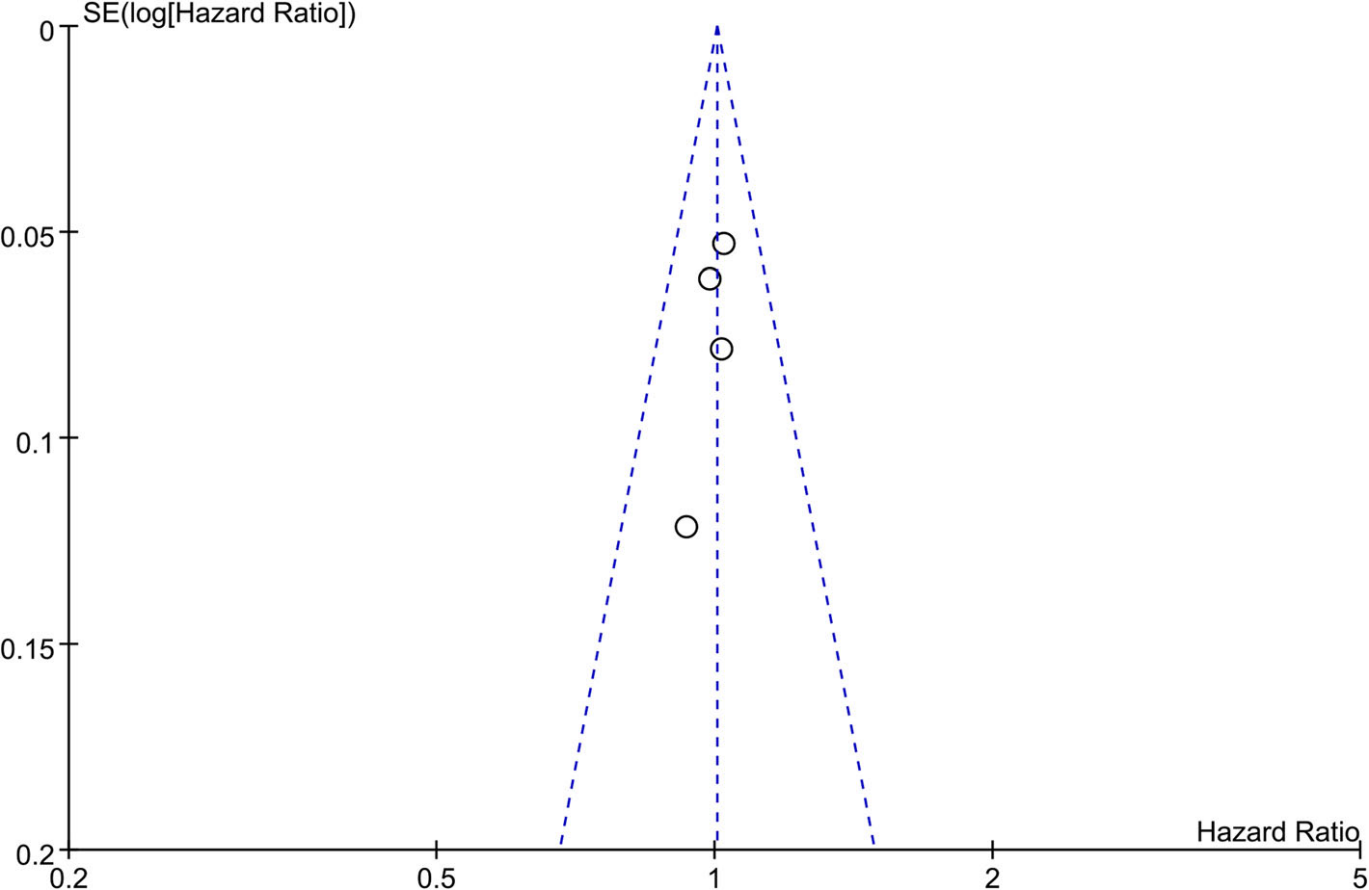
Funnel plot for the detection of publication bias. The funnel plot of the studies that evaluated the significance of SL during debulking surgery for ovarian cancer seems to be symmetrical

The results of the sensitivity analyses for OS, PFS, and postoperative complications revealed that none of the included studies alone had an obvious impact on the direction or magnitude of the outcomes. Publication bias was evaluated using funnel plots, and the shape of the funnel plot did not provide evidence of visible asymmetry (Fig. 5**)**. Therefore, the present results are statistically steady and robust.

## Discussion

For several decades, considerable debate has focused on whether pelvic and aortic lymph nodes should be systematically removed during ovarian cancer surgery. In this meta-analysis, we found that no significant differences in OS and PFS were observed between the SL and USL groups. It indicated that patients with ovarian cancer did not benefit from SL. In contrast, SL increased the risk of postoperative complications, hence, it resulted in treatment burden and harm to patients.

The results of our meta-analysis contradict the findings of many previous retrospective studies [10, 25–29] These analyses including large numbers of patients have suggested a benefit of lymphadenectomy [10, 25–29], but they are pone to several biases. For example, the decision as to which patients underwent radical cytoreductive procedures including SL was determined by the operating surgeon’s preference. Because SL is a procedure with a considerable treatment burden, surgeons would perform SL more frequently in younger and fitter patients, whereas patients with poor performance status are more likely in a USL group. This selection bias is inherent in retrospective studies and is difficult to avoid. And it may lead to the survival advantage in SL group.

Besides, our findings are not consistent with the previous meta-analyses. In 2010, Kim et al. [15] performed a meta-analysis comparing the effect of SL and USL on OS. And it showed that SL was efficient for improving OS in all-stage disease. Gao et al. [16] also conducted a meta-analysis in 2014, which confirmed the improvement of OS in the SL group. Besides, in 2016, another meta-analysis by J Zhou et al. [17] also demonstrated an increase in OS with SL, but no difference was found in PFS between the groups. The discrepancy between the previous meta-analyses and ours may due to differences in the included articles. In their meta-analyses, most of the studies included were observational ones, which have lower inferential strength than RCTs. And the number of subjects in these included RCTs was not large, which is not sufficient to describe the role of SL in patients with ovarian cancer. In addition, they used OR or RR to evaluate the role of SL in ovarian cancer which didn’t take time-point into consideration. In our study, we used HR instead to calculate both OS and PFS which would be much better. Besides, we also evaluated the postoperative complications which were not described in previous meta-analyses but important to weigh the pros and cons of SL.

The present meta-analysis showed that women in the SL group had more postoperative complications. In all four RCTs included, it was showed that SL significantly prolonged operating time, increased blood loss and the percentage of patients requiring blood transfusion. Because lymphadenectomy is always in close proximity to large vascular, the threat of intraoperative hemorrhage is always present. The systematic pelvic and especially para-aortic lymphadenectomy are considered to be the most complex and challenging technical requiring a higher surgical expertise and skill to avoid unnecessary morbidity. However, even in the hands of experienced surgeons, surgical morbidity related to lymphocyst formation, vascular events, ileus, and prolonged hospitalization may complicate a patient’s journey. And the less frequent complications such as injury to nerves, ureters and bowels may also impact the quality of patients’ life [7, 30].

It is well known that the prognosis of early ovarian cancer (EOC) is strikingly different from advanced ovarian cancer (AOC), EOC has a 10-year survival rate of over 80% [31], whereas AOC has a 5-year survival rate of approximately 30% [32]. So we discuss them separately. In EOC, SL is required for accurate staging and adequate treatment. According to the FIGO, the presence of lymph node involvement in the early stages of ovarian cancer raises the disease stage to 3A1 and requires adjuvant therapy. In addition, accurate staging in very early-stage disease may prevent unnecessary postoperative chemotherapy. Besides, SL has a proven prognostic value, lymph node metastasis is related to poor prognosis. Above all, although the effect of SL on survival is still not clear, it is paramount in the management of EOC. In AOC, the role of lymphadenectomy is therapeutic with the purpose of removing as much tumor as possible. Theoretically, the pharmacologic sanctuary hypothesis suggests that nodal metastases of ovarian cancer may be less sensitive to systemic chemotherapy due to diminished blood supply [33]. And it further implies that SL may be a favorable prognostic factor in patients with AOC who have an increased risk of occult lymph node metastasis [34]. Actually, the randomized LION trial showed patients with AOC do not benefit from SL, but experience a significantly higher rate of intraoperative and postoperative complications [18]. Thus, it seems that there is no reason to perform lymphadenectomy in this subgroup of patients.

Several limitations of our study should be considered. Firstly, heterogeneity may be present because the definition of NSL was diverse among the included articles. Additionally, the diameters of residual tumors were different, including macroscopically complete resection and up to 1 cm. Secondly, although we attempted to perform an extensive literature search to obtain all published studies, there were only 4 relevant RCTs. For this reason, we were unable to conduct subgroup analyses with regard to cancer stage, histological type, patient age or country of origin. Thirdly, we were not able to perform a search of unpublished studies, studies with negative results were less likely to be published, and thus the results of our study were limited by the inclusion of published data only. Finally, only English-language studies were included in this meta-analysis which may have introduced a language bias. However, despite these limitations, the quality of evidence was high which was about the survival impact of SL and its risk of complication.

## Conclusion

The present findings showed that SL during optimal debulking surgery for ovarian cancer was not associated with better outcomes than USL and was associated with a higher incidence of postoperative complications. SL should be reconsidered as a standard practice during optimal debulking surgery for ovarian cancer, and more well-designed studies are needed.

## Data Availability

All data generated or analyzed during this study are included in this published article [and its supplementary information files].

## Abbreviations

RCT: Randomized controlled trial
OS: Overall survival
PFS: Progression-free survival
HR: Hazard ratio
CI: Confidence interval
RR: Risk ratio
SL: Systematic lymphadenectomy
USL: Unsystematic lymphadenectomy
FIGO: International Federation of Gynecology and Obstetrics
LION: Lymphadenectomy in Ovarian Neoplasms
PRISMA: Preferred Reporting Items for Systematic Reviews and Meta-Analyses
EOC: Early ovarian cancer
AOC: Advanced ovarian cancer
